# Immunoadjuvant Activity of Fucoidans from the Brown Alga *Fucus evanescens*

**DOI:** 10.3390/md18030155

**Published:** 2020-03-11

**Authors:** Tatyana A. Kuznetsova, Tatyana P. Smolina, Ilona D. Makarenkova, Lydmila A. Ivanushko, Elena V. Persiyanova, Svetlana P. Ermakova, Artem S. Silchenko, Tatyana S. Zaporozhets, Natalya N. Besednova, Lydmila N. Fedyanina, Sergey P. Kryzhanovsky

**Affiliations:** 1 Somov Institute of Epidemiology and Microbiology, Vladivostok 690087, Russia; tsmol@mail.ru (T.P.S.); ilona_m@mail.ru (I.D.M.); l.iva_57@mail.ru (L.A.I.); helen-pers@yandex.ru (E.V.P.); niiem_vl@mail.ru (T.S.Z.); besednoff_lev@mail.ru (N.N.B.); 2 G.B. Elyakov Pacific Institute of Bioorganic Chemistry, Vladivostok 690022, Russia; swetlana_e@mail.ru (S.P.E.); artem.silchencko@yandex.ru (A.S.S.); 3 Far Eastern Federal University, School of Biomedicine, Vladivostok, 690922, Russia; fedyanina.ln@dvfu.ru (L.N.F.); kryzhanovskii.sp@dvfu.ru (S.P.K.)

**Keywords:** adjuvants, vaccines, fucoidans, innate and adaptive immunity

## Abstract

The study presents the results of a comparative evaluation of the effect of structural modifications of fucoidans from the brown alga Fucus evanescens (native, highly purified product of fucoidan enzymatic hydrolysis, a new regular 1→3;1→4-α-L-fucan, sulphated mainly at C2 and acetylated at C4 of the fucose residue) on the effector functions of innate and adaptive immunity cells in vitro and in vivo. Using flow cytometry, we found that all examined fucoidans induce the maturation of dendritic cells, enhance the ability of neutrophils to migrate and adhere, activate monocytes and enhance their antigen-presenting functions, and increase the cytotoxic potential of natural killers. Fucoidans increase the production of hepatitis B virus (HBs) specific IgG and cytokine Th1 (IFN-γ, TNF-α) and Th2 (IL-4) profiles in vivo. The data obtained suggest that in vitro and in vivo adjuvant effects of the products of fucoidan enzymatic hydrolysis with regular structural characteristics are comparable to those of the native fucoidan. Based on these data, the products of fucoidan enzymatic hydrolysis can be considered as an effective and safe candidate adjuvant to improve the efficacy of prophylactic and therapeutic vaccines.

## 1. Introduction

A large number of publications are devoted to the study of the biological activity of fucoidan from the brown alga *Fucus evanescens*. The results of these studies are summarized in our work [1]. These data indicate that the fucoidan from *F. evanescens* has high biocompatibility, lack of toxicity, safety for the macroorganism, and has various experimentally and clinically proven pharmacological effects, including antitumor, anticoagulant, antimutagenic activities, and immunostimulatory, antiinfective, and antioxidant properties, providing great potential for designing drugs for biomedical use on the fucoidan basis.

**Figure 1:**
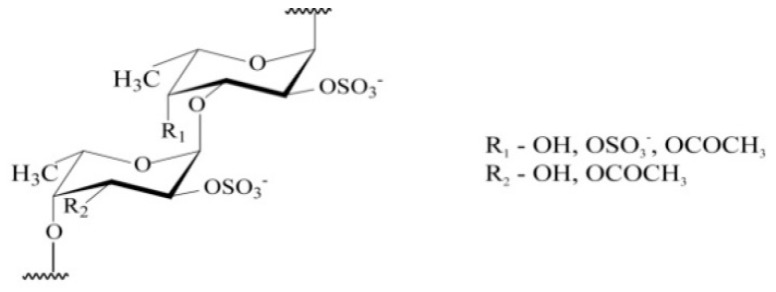
Structure of fucoidan from *Fucus evanescens.*

However, no fucoidan has been declared as a drug. The reason is that the structural diversity of fucoidans is extremely large. Fucoidans represent the family of fucose-containing homo- and heteropolysaccharides from polysaccharides with a high content of uronic acids and low fucose and sulfate contents, for example, from *Hizikia fusiforme* alga, for practically pure α-L-fucans with the main component of polysaccharide-fucose (*F. evanescens*). Except for fucose, these polysaccharides can contain minor amounts of other monosaccharides (galactose, mannose, xylose, glucose) and also sulfates, uronic acids, acetyl groups, and protein [2,3,4,5]. In particular, fucoidan from *F. evanescens* has a similar l-fucosyl backbone of alternating α (1→4) and α (1→3) l-fucosyls with sulphate substitution at C2. An additional sulphate may occupy position 4 in some of the α (1→3)-linked fucosyls, and the remaining hydroxyl groups may be randomly acetylated [3,4] (Figure 1).

The figure shows the structure of fucoidan from *F. evanescens*: fucoidan has a similar l-fucosyl backbone of alternating α (1→4) and α (1→3) l-fucosyls with sulphate substitution at C2. An additional sulphate may occupy position 4 in some of the α (1→3)-linked fucosyls, and the remaining hydroxyl groups may be randomly acetylated.

In order to establish the structure of polysaccharides, the most promising approach is based on the use of enzymatic transformation of fucoidans that can be extremely useful not only for the establishment of structural features, but also for access to biologically active fragments [6,7]. As an example, the product of enzymatic hydrolysis of fucoidan from *F. evanescens* has been obtained [6].

The present study aimed to comparatively evaluate the effect of fucoidans from the brown alga *F. evanescens* (native, highly purified product of fucoidan enzymatic hydrolysis) on effector functions of innate and adaptive immunity cells in vitro and in vivo.

## 2. Results

### 2.1. The Effect of Fucoidans on the Maturation of Dcs Generated from Mice Bone Marrow

We revealed that all fucoidans (F1-3) induce the maturation of dendritic cells (DCs) generated from the bone marrow of mice, as evidenced by a pronounced increase in the expression of CD83 (the terminal differentiation marker) reducing the expression of CD34 (the marker of immature DCs) compared to the negative control (*p* < 0.01). Under the fucoidan action, expression of the activation marker (CD38) and adhesion molecule (CD11c) increased compared to the negative control (*p* < 0.05). The expression of costimulatory (CD80 and CD86) and antigen-presenting molecules (MHC class II) increased compared to the negative control (*p* < 0.05). The effect of F3 on the expression of the terminal differentiation marker (CD83) was more pronounced compared with F1 and F2 (*p* < 0.05). The presence of the CD14 monocyte marker on DCs indicates heterogeneity of the population. A decrease in the expression of CD14 after treatment by fucoidans (*p* < 0.05) against an increase in CD83 (*p* < 0.01) confirms the differentiation of cells into DCs (Table 1).

It should be noted that the effect of fucoidans on the maturation of DC was comparable to the action of the classical maturation inducer, TNF-α, used as a positive control. Generated DCs have the phenotypes CD34^+^/^−^; CD83^+^; CD11c^+^; CD38^+^; CD80^+^; CD86^+^; MHCII^+^; CD14^+/-^ (Table 1).

### 2.2. The Effect of Fucoidans on CD Molecule Expression in Peripheral Blood Human Innate Immunity Cells (Neutrophils, Monocytes, NK-Cells)

We revealed the pronounced changes in the functional state of neutrophils incubated with fucoidans: an increase in the expression levels of the activation molecules CD69 and CD14, an increase of the expression levels of adhesion molecules CD11b, which belong to the β2-integrin family, and a decrease the expression levels of CD62L molecules from the L-selectin family compared to the control (*p* < 0.01). A comparison of measurement results indicates that the expression of CD14 and CD11b during incubation of neutrophils with F1 was lower (*p* < 0.05), and CD62L molecules-higher (*p* < 0.05) compared to F3 (Table 2).

**Figure 2:**
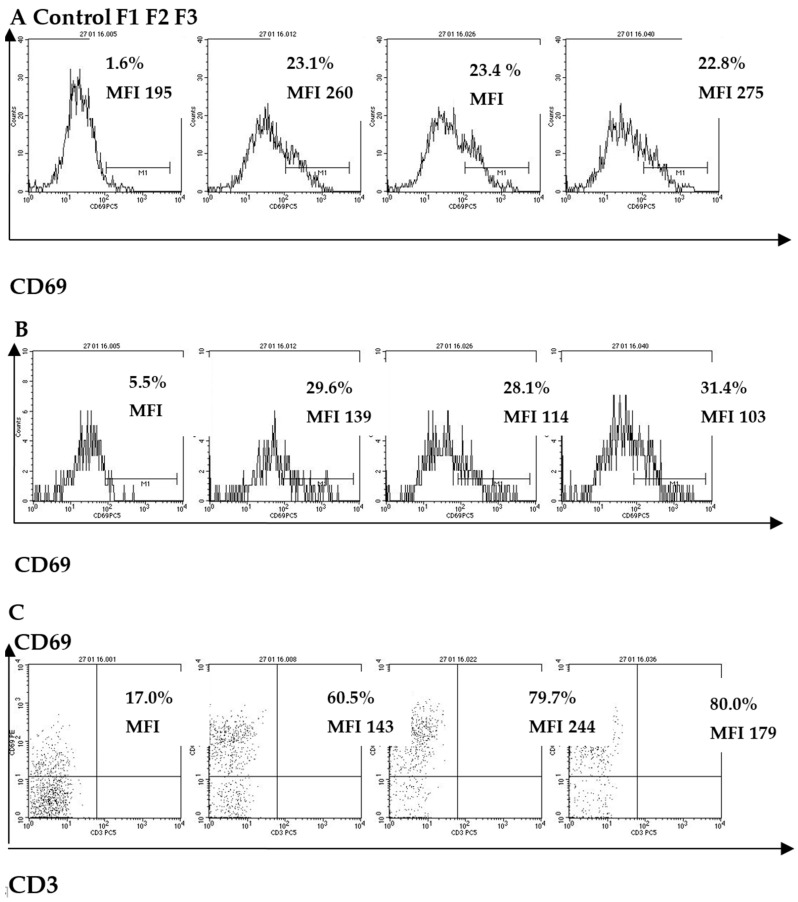
The effect of fucoidans from *F. evanescens* on CD69 molecule expression on peripheral blood human innate immunity cells: (**A**) neutrophils, (**B**) monocytes, (**C**) NK-cells.

All studied fucoidans (F1-3) equally and significantly, compared to the control, increased the expression of the early activation molecule CD69, recorded both in terms of the percentage (%) of monocytes expressing CD69 such as MFI (*p* < 0.05) (Table 2, Figure 2). The activation of monocytes after treatment by fucoidans was accompanied by an increase in the expression level of HLA-DR (the antigens of the main histocompatibility complex) (*p* < 0.05) and a significant increase in the expression of CD83 (the main marker of mature DC) compared to the control (*p* < 0.05) (Table 2).

All fucoidans increased the expression of CD69 on NK-cells compared to the control (*p* < 0.05), as well as the number of NK-cells expressing CD25 (*p* < 0.01). Fucoidans F1 and F2 increased the expression of CD8 (by measuring MFI) compared to the control (*p* < 0.05), and F1 and F3 increased the content of NK cells expressing CD107a compared to the control (*p* < 0.05) (Table 2).

The data of MFI (mean fluorescence intensity) and average percentage (%) of cells expressing CD molecules are presented as Me (median) and min-max; * *p* < 0.05; ** *p* <0.01 compared to the control, Wilcoxon-test.

The figure shows the results of flow cytometry analysis of CD69 marker expression on peripheral blood cells (neutrophils, monocytes, NK-cells) of healthy volunteers. The time of incubation of fucoidans (in concentration, 100 µg/mL) with whole blood was 24 h. Control-PBS without fucoidan; F1: native fucoidan in complex with polyphenols; F2: highly purified fucoidan; F3: the product of fucoidan enzymatic hydrolysis. The surface expression of CD69 on innate immunity cells (neutrophils, monocytes, NK-cells) was assessed in whole blood samples using immunofluorescent staining with appropriate monoclonal antibodies (MAbs) and the appropriate isotypic controls. A minimum of 10000 cells were analyzed for each sample. Stained cells were analyzed using a BD FACS Calibur flow cytometer and CellQuest software (Becton Dickinson, USA). The results are shown as the percentage of CD69 molecules (%) and fluorescence intensity (MFI).

### 2.3. The Effect of Fucoidans on the Expression of Activation Molecules on Peripheral Blood T- and B-Cells

All fucoidans increased the expression of CD69 molecules on the T-cell surface compared to the control (*p* < 0.05). Fucoidan F1 contributed to an increase in the expression of CD25, HLA-DR, and CD71 molecules on T-cell membranes compared to the control (*p* < 0.05). Fucoidans F2 and F3 had no effect on the expression of the activation markers CD25, HLA-DR, and CD71. It is possible that increased expression of these markers will be observed with an increase in the incubation time of fucoidans with cells. Fucoidans (F1-3) significantly enhanced the expression of markers CD69, CD25, CD86, CD71, and TLR2 on B-cell membranes compared to the control (*p* < 0.05); the effects of F1-3 on B-cells were comparable (Table 3).

### 2.4. Adjuvant Effect of Fucoidans on the Hb-Specific Immune Response and Cytokine Production In Vivo

**Figure 3:**
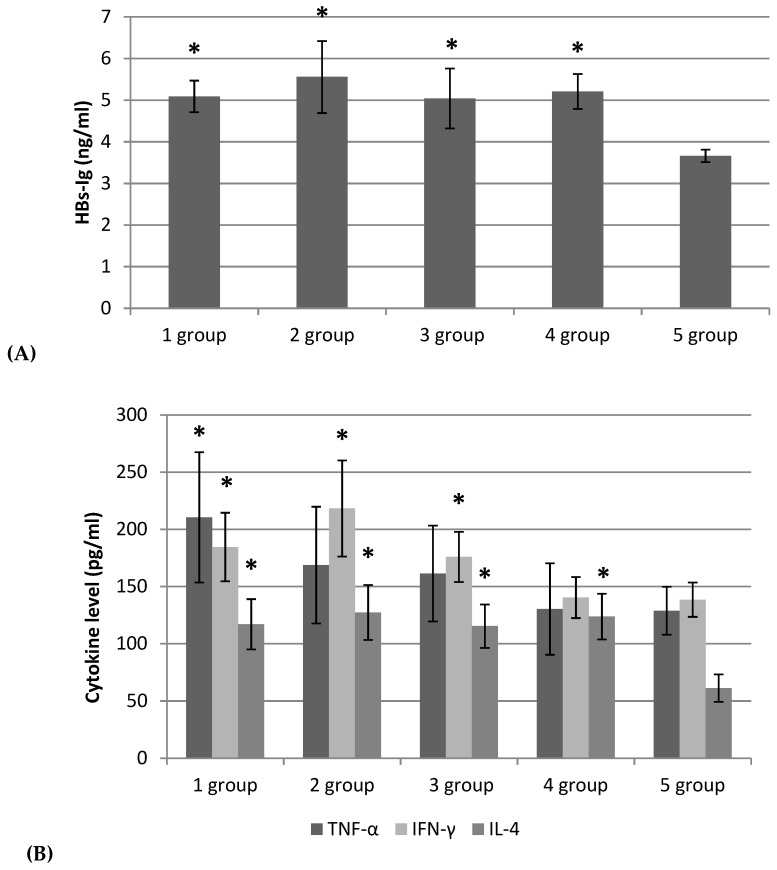
The level of serum IgG antibodies (**A**) and cytokines (TNF-α, IFN-γ, IL-4) (**B**) after mice BALB/c immunization with HBs-AG.

HB-specific antibody analysis showed that 4 weeks after immunization, all examined samples of fucoidans as well as aluminum hydroxide gel (positive control adjuvant) (1–4 groups) induced a more pronounced immune response compared to the control 5 group (HBs-AG without adjuvant) (*p* < 0.05). There were no differences in specific HBs-IgG levels in animals groups 1–3 vaccinated with HBs-AG and fucoidans compared to group 4, where aluminum hydroxide gel was used as an adjuvant (Figure 3A).

To establish the involvement of T-cells and specifically Th1/Th2 polarization of immune responses which induced immunization by HBs-AG, we evaluated cytokine levels in sera. Fucoidans (F1-3) in the vaccine with HBs-AG in groups 1-3 elicited cytokine (IFN-γ, TNF-α, IL-4) levels higher than the control group (5) (*p* < 0.05). The cytokine levels were comparable in groups 1–3 (Figure 3B). The IFN-γ/IL-4 ratios in groups 1–3 (vaccine with HBs-AG with fucoidans F1-3) were equal, i.e., 1,6; 1,7; and 1,5, respectively, to group 4 (vaccine with HBs-AG with aluminum hydroxide gel)-1,1 (Figure 3B).

The figure summarizes results obtained in experiments with a total of 60 mice. Female BALB/c mice were randomized into five groups, immunized with subcutaneous vaccines as follows: group 1: HBs-AG (Abcam Limited, United Kingdom) with fucoidan (F1); group 2: HBs-AG with fucoidan (F2); group 3: HBs-AG with the production of enzymatic hydrolysis of fucoidan (F3); group 4: HBs-AG with aluminum hydroxide gel (Sigma, A8222); group 5: HBs-AG with phosphate buffer solution (PBS). Mice were immunized with 1 μg/mouse HBs-AG alone or together with 50 mg/kg body weight of fucoidans (F1, F2, F3) on day 0 and 14. A suspension of HBs-AG with fucoidans or with aluminum hydroxide gel was prepared by mixing or adsorbing aluminum gel for 1 h. Blood serum was obtained 4 weeks after immunization of mice. The total IgG antibodies were detected by a Mouse Hepatitis B surface Antibody (HBsAb) ELISA kit (Blue Gene Biotech, China). The serum levels of TNF-α and IFN-γ were measured by ELISA kits (BD Biosciences OptEIATM Set Mouse; USA), and IL-4 by an ELISA kit (BioLegend, London, UK). Each bar represents mean ± SD (a pool of 2 mice; n = 6). Levels of significance of groups 1–4 versus the control group (5): * *p* < 0.05, Student’s t-test.

## 3. Discussion

Vaccine adjuvants are necessary for successful immunization. Numerous experimental data indicate that sulfated polysaccharides from brown algae (fucoidans) demonstrate properties of vaccine adjuvants for enhancement of anti-infective and antitumor immune responses. Over the last years, there were a number works devoted to the ability of fucoidans to enhance the production of specific antibodies, both to model and to various bacterial and viral antigens as well as to stimulate the T-cell response in vivo. Fucoidan from *F. vesiculosus* increased the level of specific antibodies to ovalbumin (OVA) and the T-cell response in experimental animals and also stimulated the maturation of DC. In mice receiving an anticancer vaccine together with fucoidan, considerable strengthening of functional activity of spleen lymphocytes in comparison with the animals receiving the same vaccine without fucoidan was observed. Animals of the experimental group were protected from development of the tumor induced by B16-OVA melanoma cells. Authors noted more active proliferation of T-lymphocytes participating in the destruction of tumors, enhancement of number of T-cells in the spleen producing IFN-γ and TNF-α-signaling cytokines of Th1, and an increase in serum levels of these cytokines under the influence of fucoidan [8]. The injection of mice with fucoidans from brown algae *Ascophyllum nodosum*, *Macrocystis pyrifera*, *Undaria pinnatifida,* and *F. vesiculosus* led to strengthening of the production of IgG1 and IgG2a OVA-*specific* immunoglobulins and also the T-cell response and generation of T-memory cells [9]. Fucoidans from algae *Grateloupia filicina*, *Ulva pertusa,* and *Sargassum qingdaoense* differing in chemical structure showed antiviral activity towards avian influenza virus H9N2 subtype and immunomodulatory activity both in vitro and in vivo [10], and fucoidan from *Sargassum pallidum* strengthened specific humoral and cellular immune responses in vaccine antigens of the combined vaccine against Newcastle disease, infectious bronchitis, and avian influenza in experiments on chickens [11]. The adjuvant activity of fucoidan from *F. vesiculosus* towards a vaccine strain of *Mycoplasma hyopneumoniae* was revealed [12]. This polysaccharide promoted strengthening of the production of specific antibodies to the studied antigen and also an expression of the molecules MHC II, CD25, and CD69 on spleen cells and CD19 on B-lymphocytes. Treatment of mice BALB/c spleen cells with fucoidan together with a vaccine of the *Bordetella bronchiseptica* antigen led to increased TNF-α production at lower doses of the antigen, which can be the mechanism providing the stimulating influence on the functional activity of effector cells of innate and adaptive immunity, strengthening the vaccination efficiency [12]. Based on the results, the authors conclude that fucoidans can function as adjuvants in various type of preventive and therapeutic vaccines.

The defining property of vaccines adjuvants is their ability to stimulate (modulate) the innate and adaptive immunity system.

Research on the immunostimulatory (immunomodulatory) properties of fucoidans in various experimental models showed that the main target for fucoidans are effector cells of innate immunity: monocytes/macrophages [13,14], neutrophilis [15], and NK-cells [9,16,17,18]. Due to the ability of fucoidans to stimulate cytotoxic effects of NK, the potential effect of fucoidans as adjuvants in the anticancer immune response is under intensive study [18]. It has been reported that fucoidans promote DC maturation [8,16,19,20].

Understanding the mechanisms of adjuvant action provides important information on how innate immunity influences the development of adaptive immunity, helping in the rational selection of vaccine adjuvants. In this regard, the processes of activation and differentiation of cells of innate and adaptive immunity require special attention when studying the adjuvant activity of fucoidans.

The objects of our study were fucoidans (native, highly purified products of fucoidan enzymatic hydrolysis) from brown alga *F. evanescens,* belonging to the family *Fucaceae,* genus *Fucus.* As a result of the research, the activating effects (F1-3) on the innate and adaptive immunity cells were detected, as evidenced by the increased expression of activation markers. In particular, fucoidans induce the maturation of DCs, enhance the ability of neutrophils to migrate and adhere, activate monocytes and enhance their antigen-presenting functions, and increase the cytotoxic potential of NK cells in vitro.

Mature DCs are characterized by an increase in the expression of molecules involved in antigen presentation and promote the formation of an immune synapse for signal transmission from antigen presenting cells to T lymphocytes and differentiation of activated T cells into T-effector cells [19,20,21,22]. Some studies have shown that TLR activation in the DC presenting the antigen is critical for CD4^+^ T cell activation and Th1 cell differentiation [23,24].

A significant increase in monocytes expressing CD83, the main marker of mature DCs, after treatment by fucoidans, indicates the readiness of these cells for differentiation into DCs. The increase in the expression of the activation marker (CD38) and adhesion molecule (CD11c) indicates the ability of matured DCs to interact with T-lymphocytes and increase costimulatory (CD80 and CD86), antigen-presenting molecules (MHC class II), indicating the ability of DCs to further activate naive T-cells [24,25].

The effect of fucoidans on neutrophils leads to a change in their expression levels: an increase in activation markers CD69, CD14, and adhesion molecules CD11b, which is evidence of the activation of neutrophils, and enhances their ability to migrate and adhere.

All studied fucoidans increased the expression of CD69 and the number of NK-cells expressing CD25, which characterizes cell activation and enhanced proliferation [26,27]. In addition, F2 enhanced the expression of HLA-DR. Fucoidans also increased the expressions of CD8 and CD107a. Increased expression of markers CD8 and CD107a indicates degranulation and, consequently, an increase in the cytotoxic (killer) activity of NK cells after treatment by the studied fucoidans.

Also, the studied fucoidans (F1-3) enhanced the expression of activation markers on T-cells and on B-cells. The increase in the expression of CD69, CD25, HLA-DR, and CD71 molecules on T-cells facilitates the initiation of signaling events that regulate the entry of dormant cells into the cell cycle and has an effect on the formation of specialized T-lymphocytes for memory that effectively form the late phase humoral immune response.

The increased activation molecule expression on T- and B-lymphocytes under the fucoidans influence contributed to the enhancement of humoral immunity, providing various stages of activation of B-lymphocytes.

The immune response to various AG requires the participation of different types of immune responses [28]. Thus, the Th1 response correlates with the induction of cellular immunity, which is necessary to protect against intracellular infectious agents (bacteria, protozoa) and is characterized by the production of cytokines IFN-γ, TNF-β, and IL-2 and enhanced production of IgG2a, IgG2b, and IgG3 in mice [29]. Th2, which controls humoral immunity, is effective for protection against most bacterial and several viral infections and is characterized by the formation of cytokines IL-4 and IL-10 and enhanced production of IgG1 [28,30,31,32]. The adjuvant determines the direction of the response generated by antigens along the Th1 or Th2 pathway [30].

Fucoidans (F1-3) in the vaccine with HBs-AG induced a more pronounced immune response and cytokine Th1- (IFN-γ, TNF-α) and Th2- (IL-4) production compared to the control (HBs-AG without fucoidan as an adjuvant). Thus, fucoidans stimulated a specific humoral immune response and had a regulatory effect on cytokine production (Th1 and Th2) in vivo. As known, the IFN-γ/IL-4 ratio is a valuable indicator of the deviation or amplification of the Th1 response. In the case of hepatitis B vaccine, the best model of the immune response is a mixture of Th1/Th2 responses [33], but alum adjuvants shift the immune responses towards Th2 immunity, which is responsible for antibody production [34]. Our findings demonstrated that the IFN-γ/IL-4 ratio in the vaccine with HBs-AG and fucoidan (F1-3) was higher (1,6; 1,7; 1,5 respectively) than that measured for HBs-AG with aluminum hydroxide gel (1,1). The results of the IFN-γ/IL-4 ratio of fucoidan-based vaccines were considerable and will improve the efficacy of hepatitis B vaccines.

These findings positively characterize fucoidans from *F. evanescens* as potential adjuvants, which will promote the activation of innate and adaptive immunity cells and enhance antigen-specific B-cell reactions, increasing the immunogenicity of vaccines.

Among the studied polysaccharides of particular interest is the product of fucoidan enzymatic hydrolysis (F3), which has standard structural characteristics: regular 1→3;1→4-α-L-fucans, sulphated mainly at C2 and acetylated at C4 of the fucose residue. The activating effects of F3 on the effector functions of innate and adaptive immunity cells are comparable to those of the native fucoidan (F1). However, unlike the latter, the product of fucoidan enzymatic hydrolysis has a lower molecular weight and is characterized by a regular reproducible structure. Along with high biocompatibility and a lack of toxicity, it can be considered an effective and safe candidate adjuvant to improve the efficacy of prophylactic and therapeutic vaccines. However, the pathways of immune regulation and the mechanisms of adjuvant effects require further clarification.

## 4. Materials and Methods

### 4.1. Chemical Characteristics of the Fucoidans

Fucoidans from the brown alga *Fucus evanescens* were studied (Table 1): F1, native (crude) fucoidan in complex with polyphenols [35]; F2, highly purified fucoidan [36]; F3, the product of enzymatic hydrolysis of fucoidan [6] (Table 4). According to the results of nuclear magnetic resonance analysis, this fraction represents regular polysaccharides, with the following structure of repeating units: [→3)-α-L-Fucp(2,4-SO^−^_3_)-(1→4)-α-L-Fucp(2-SO^−^_3_)-(1→]_n_. The polysaccharide is sulfated mainly at C2 and to a lesser degree at C4. The acetyl groups occupy the free position at C4 [6].

The table summarizes the data of fucoidans from the brown alga *Fucus evanescens*: F1, native (crude) fucoidan in complex with polyphenols; F2, highly purified fucoidan; F3, the product of enzymatic hydrolysis of fucoidan. According to the results of nuclear magnetic resonance analysis, this fraction represents a regular polysaccharide, with the following structure of repeating units: [→3)-α-L-Fucp(2,4-SO^−^_3_)-(1→4)-α-L-Fucp(2-SO^−^_3_)-(1→]_n_. The polysaccharide is sulfated mainly at C2 and to a lesser degree at C4. The acetyl groups occupy the free position at C4.

The endotoxin levels in fucoidans were evaluated using a Limulus amebocyte lysate (LAL) assay kit Kinetic QCL-1000 (Cambrex Bio Science, CIIIA) with methods of gas-liquid chromatography (GLC) and GLC-mass spectrometry (chromatograph: Agilent 6850 Serients GL Sistems, Germany, mass spectrometer: Hewlett Packard 5973, USA). The test results indicate the absence of endotoxin in the composition of fucoidans.

### 4.2. Peripheral Blood Cells

The study was performed with peripheral blood of healthy volunteers (n = 5) (Protocol № 1 approved Research Ethics Committee of G.P. Somov Research Institute of Epidemiology and Microbiology 14.05.2018) in blood collection tubes with heparin. The blood was cultured in Roswell Park Memorial Institute medium1640 (RPMI-1640 medium) with 10% bovine serum albumin (BSA), 2 mmoL of L-glutamine, and 20 mg of gentamicin in a CO_2_ incubator at 37 °C for 24 h. Fucoidans (F1-3) were added at a concentration 100 μg/mL.

### 4.3. Flow Cytometry Analysis

The surface expression of CD69, CD25, CD14, HLA DR, CD8, CD62L, CD11b, CD56, CD107a, CD71, CD83, CD86, CD3, CD20, and TLR-2 on human cells (neutrophils, monocytes, NK-cells, T- and B-lymphocytes) was assessed in whole blood samples using immunofluorescent staining with appropriate monoclonal antibodies (MAbs) and the appropriate isotypic controls. Stained cells were analyzed using a BD FACS Calibur flow cytometer and CellQuest software (Becton Dickinson, USA). Lymphocyte, granulocyte, and monocyte subpopulations were first gated according to forward and side scatter. B-cells were then identified as CD20-expressing cells, and T-cells were then identified as CD3-expressing cells. Monocyte analyses were carried out using forward and side scatter parameters combined with CD14-positively stained cells. NK-cell gating was carried out using CD56-fluorescence in the CD3-negative lymphocyte area. To exclude non-living cells from gates, cell staining with 7-AAD (7-aminoactinomycin D) was used. A minimum of 10000 cells were analyzed for each sample. The results are shown as the average percentage (%) of cells expressing corresponding markers or mean fluorescence intensity (MFI), which reflects the number of molecules expressed on the cell membrane.

Monoclonal antibodies (MAbs): CD69–PC7 (A80710); CD69–PE (IM1943U), CD25–PE (AO 7774); CD14–FITC (6604110); HLA DR–PE (IM1639); CD8–FITC (AO 7756); CD62L–FITC (IM 1231U); CD11b–PE (IM 2581U); CD56–APC (IM 2474); CD71–PE (IM2001U); CD83–PE (IM 2218U); CD86–PE (IM2729U); CD3–PC5 (A66327); CD20–FITC (AO7772) were obtained from Beckman Coulter, USA; CD107a–PE (12-1079-42) and TLR-2–APC (17-9922-42) (CD282) were obtained from eBioscience, Austria.

### 4.4. Mice and Ethics Statement

Female BALB/c mice (6–8 weeks old) were obtained from the nursery of the Scientific and Research Center for Biomedical Technologies “Andreyevka” (Moscow region) and kept under pathogen-free conditions. All experiments were carried out under the guidelines of the European Convention for Protection of Vertebrate Animals used in Experimental and other Studies. Cets No.: 123 Strasbourg, 18.03.1986. Mice were sacrificed by ether euthanasia. All animal procedures were approved by the Research Ethics Committee of G.P. Somov Research Institute of Epidemiology and Microbiology (Protocol Number 1 approved 14.05.2018). Cervical dislocation was adopted to provide mice with a fast and painless death.

### 4.5. DC Generation

DC was obtained from bone marrow cells of 24 BALB/c mice. Animal bone marrow was homogenized in RPMI-1640 medium (Sigma, USA), precipitated three times by centrifugation (250 g × 5 min), and transferred to enriched culture medium (10^6^ cell /mL in RPMI–1640 medium with the addition of 100 μg/mL gentamicin sulfate and 10% thermally activated fetal calf serum, containing recombinant growth factors GM-CSF and IL-4 (80 and 20 ng/mL) (eBioscience, USA). Cells were cultured at 37 °C in a 5% CO_2_ incubator. On the third day, cytokine re-stimulation was performed. The medium was changed on the sixth day, adding to the experimental vials of the population of immature DC maturation inducers: fucoidan samples (100 μg / mL), and as the second positive control, the classical maturation inducer-TNF-α (20 ng/mL) (eBioscience, USA). The control was immature DC (n–DK). Cells were cultured for 48 h at 37 °C in a 5% CO_2_ incubator.

Flow cytometry analysis DC was evaluated using a Cytomix FC–500 (Beckman Coulter, USA) with MAbs (eBiosciences, USA) labeled with a fluorochrome for the designated markers: CD34–FITC, CD83–FITC, CD14–PE, CD11c–FITC, CD38–PE, CD80–FITC, CD86–FITC, MHC II–FITC (eBioscience, USA).

MAbs: CD34–FITC (clone RAM 34, №. 11-0341-82), CD38–FITS (clone 90, № 11-0381-82), CD14–PE (clone Sa2-8, № 12-0141-82), CD11c–FITS (clone 418, № 11-0114-81), CD38–PE (clone Michel-17, № 12-0831-80), CD80–FITS (clone 16-10A1, № 11-0801-82), CD86–PE (GL-1, № 120862-81), MHCII–FITS (clone NIMR-4, № 11-5322-82), (eBioscience, CIIIA).

### 4.6. Immunization of Mice with Recombinant Hepatitis B Virus Surface Antigen (Hbs-AG) (Abcam Limited, United Kingdom) and Blood Sampling

Female BALB/c mice were randomized into 5 groups (12 mice in each), immunized subcutaneously with the vaccine as follows: group 1: HBs-AG with fucoidan (F1); group 2: HBs-AG with fucoidan (F2); group 3: HBs-AG with the product of enzymatic hydrolysis of fucoidan (F3); group 4: HBs-AG with aluminum hydroxide gel (Sigma, A8222); group 5: HBs-AG with phosphate buffer solution (PBS). BALB/c mice were immunized with 1 μg/mouse HBs-AG alone or together with 50 mg/kg fucoidans (F1-3) on day 0 and 14. A suspension of HBs-AG with fucoidans or with aluminum hydroxide gel was prepared by mixing or adsorbing on aluminum gel for 1 h. A phosphate buffer solution, pH 7.2 (PBS) was used as a solvent. Blood serum was obtained from each mouse 4 weeks after immunization and stored at −70 °C until tested.

### 4.7. ELISA, Antibody Titration

The total IgG antibodies were detected by a Mouse Hepatitis B surface Antibody (HBsAb) ELISA kit (Blue Gene Biotech, China) using the manufacturer’s instructions. The results were measured on a Multiscan RC microplate reader (Labsystems, Finland) at 450 nm. Serum antibody titres were calculated in ng/mL and expressed as the mean ± SD of triplicate determinations.

### 4.8. Quantitative Detection of Cytokines in the Serum of Mice

The serum levels of TNF-α, IFN-γ were measured by ELISA kits (BD Biosciences OptEIATM Set Mouse; USA), IL-4 by ELISA kit (BioLegend, London, UK) using the manufacturer’s instructions. The results were measured on a Multiscan RC microplate reader (Labsystems, Finland) at 450 nm. The conversion of measured absorbance values to pg/mL was performed from the calibration curve generated using standards included in each kit.

### 4.9. Statistical Analysis

Statistical analysis was performed using Statistica 10.0 software package (StatSoft, Tulsa, OK, USA). The data of flow cytometry of peripheral blood human immunity cells (non-parametric analysis) are expressed as Me (median) and min-max; results were confirmed for at least five healthy volunteers in independent experiments. The significant differences in the data were analysed by a Wilcoxon-test; *p*-values < 0.05 were considered statistically significant compared to the control. Analysis of intergroup differences was performed using ANOVA.

The data of ELISA (antibody titration and detection of cytokines) are expressed as means ± SD. The experimental group of mice (1–4) was compared with the control (5) group by performing an unpaired Student t-test. Differences were considered significant where * *p* < 0.05, ** *p* < 0.01.

## 5. Conclusions

Fucoidans from the brown alga *Fucus evanescens* (native, highly purified products of fucoidan enzymatic hydrolysis) activate effector functions of innate and adaptive immunity cells in vitro and function as adjuvants to enhance HBs-specific immune responses (IgG) and cytokine Th1 (IFN-γ, TNF-α) and Th2 (IL-4) profiles in vivo.

## Figures and Tables

**Table 1:** The effect of fucoidans from *F. evanescens* on the immunophenotype of dendritic cells generated from the bone marrow of BALB/c mice.

Percentage (%) of Cells Expressing Markers	Negative Control (Immature DC-GM-CSF and IL-4)	Positive Control (TNF-α)	Fucoidans		
			F1	F2	F3
CD34	42.9 ± 4.5	6.9 ± 2.1 **	10.2 ± 0.9 **	14.3 ± 5.7 *	12.2 ± 5.6 **
CD83	8.1 ± 1.9	68.1 ± 4.8 **	53.9 ± 7.5 **	48.5 ± 6.5 **	64.7 ± 5.6 **
CD14	57.2 ± 6.2	34.7 ± 4.6 *	40.1 ± 0.7 *	34.8 ± 7.6 *	42.1 ± 5.4 *
CD11c	10.5 ± 3.7	38.5 ± 1.4 **	21.9 ± 5.9 *	27.1 ± 4.9 *	32.1 ± 4.1 *
CD38	20.2 ± 3.6	35.8 ± 5.3 *	27.9 ± 2.5 *	38.8 ± 8.8 *	37.6 ± 3.2 *
CD80	14.3 ± 4.3	51.1 ± 5.6 **	28.8 ± 4.2 *	35.7 ± 5.9 *	49.6 ± 8.6 *
CD86	15.6 ± 2.4	57.9 ± 6.7 **	41.4 ± 3.9 **	30.2 ± 7.6 *	36.1 ± 5.6 **
MHC-II	20.9 ± 1.7	78.1 ± 2.8 **	70.7 ± 7.1 **	63.4 ± 2.5 **	71.9 ± 6.3 **

The table summarizes the data of flow cytometry analysis of surface marker expression on dendritic cells (DCs) generated from the bone marrow of BALB/c mice. Each symbol represents means ± SD (a pool of 2 mice; n = 6); * *p* < 0.05; ** *p* < 0.01 compared to the negative control (immature DCs), Student’s t-test. The table summarizes results obtained from two experiments with a total of 24 mice.

**Table 2:** The effect of fucoidans from *F. evanescens* on CD molecule expression on peripheral blood human innate immunity cells.

Cell Subpopulation	CD Molecule	Results	Fucoidans			Control
			F1	F2	F3	
Neutrophils	CD69	MFI	74.3 * (48–87)	82.0 ** (51–104)	87.3 ** (49–123)	31.2 (22–49)
	CD14	MFI	52.0 * (43–82)	78.6 ** (62–101)	75.3 ** (66–94)	25.3 (18–30)
	CD11b	MFI	2621.3 * (1738–3670)	2706 * (1839–3631)	2981 * (2341–3894)	1023 (802–1264)
	CD62L	MFI	62 ** (45–78)	37.3 ** (30–45)	34.8 ** (29–44)	174 (142–211)
Monocytes	CD69	%	30 ** (22–44)	29 ** (24–45)	28 ** (23–41)	5 (3–15)
		MFI	139 * (95–146)	114 * (96–143)	103 * (89–125)	38 (23–45)
	HLA-DR	%	68 * (55–80)	77 * (69–85)	75 * (68–81)	47 (44–51)
		MFI	153 ** (118–186)	200 ** (157–241)	149 ** (113–183)	61 (48–74)
	CD83	%	4.3 * (2.0–6.6)	4.8 * (2.3–7.3)	7.2 * (4.6–9.7)	0.8 (0.6–0.9)
		MFI	3.7 * (2.2–4.2)	5.3 * (3.8–6.7)	4.9 * (3.1–4.7)	1.5 (1.1–1.8)
NK-cells	CD69	%	57.9 ** (53.6–61.9)	78.1 ** (73.2–81)	78.9 ** (74–81.2)	12.7 (8.5–20.7)
		MFI	140.4 ** (105.4–174)	220 ** (154–265)	170 ** (121–202)	42.8 (31.2–64.7)
	CD25	%	7.0 * (5.2–9.3)	6.7 * (4.5–8.7)	5.6 * (4.1–6.9)	2.8 (1.3–3.7)
		MFI	6.3 (4.8–7.5)	5.2 (3.6–6.8)	6.4 (4.9–7.3)	5.9 (3.5–6.8)
	HLA-DR	%	4.7 (3.0–6.4)	8.4 * (6.2–10.5)	4.4 (2.8–6.9)	4.2 (2.1–6.7)
		MFI	2.8 (1.3–3.9)	6.1 * (4.5–8.9)	2.2 (1.2–4.0)	2.0 (1.1–3.4)
	CD8	%	57.6 (53.4–61.2)	56.7 (51.6–59.7)	54.2 (49.3–58.9)	55.5 (49.0–58.1)
		MFI	193 * (179–207)	183 * (174–191)	163 (152–173)	159 (145–162)
	CD107a	%	2.6 * (1.8–3.9)	2.1 (1.6–3.8)	4.0 * (2.5–5.7)	1.4 (0.7–1.8)

The table summarizes the data of flow cytometry analysis of surface marker expression on human peripheral blood cells (neutrophilic leukocytes, monocytes, NK-cells) of healthy volunteers (n = 5). The time of incubation of fucoidans (100 µg/mL) with whole blood was 24 h. * *p* < 0.05; ** *p* < 0.01.

**Table 3:** The effect of fucoidan from *F. evanescens* on CD molecule expression on peripheral blood human adaptive immunity cells.

Cell Subpopu Lation	CD Molecule	Results of Measure Ment	Fucoidans			Control
			F1	F2	F3	
T-cells	CD69	%	5.8 * (3.6–7.2)	6.2 * (3.8–8.9)	6.5 * (4.1–8.8)	3.0 (1.6–5.4)
	CD25	%	9.1 * (6.2–14.1)	6.4 (4.1–8.8)	5.3 (3.1–7.8)	5.9 (3.7–7.9)
	HLA-DR	%	5.1 * (3.4–8.7)	3.8 (2.2–6.1)	3.6 (2.9–6.2)	3.4 (1.9–5.6)
	CD71	%	6.4 * (4.2–8.9)	5.4 (3.2–9.0)	4.9 (3.4–8.1	4.1 (2.1–6.9)
B-cells	CD69	%	25.1 * (18.5–28.4)	28.7 * (16.4–32.6)	24.4 * (17.9–28.6)	11.3 (6.2–14.2)
	CD25	%	6.6 ** (4.9–9.5)	5.8 * (3.3–8.2)	6.9 * (4.1–10.1)	2.3 (1.2–3.5)
	CD86	%	15.9 ** (11.8–18.6)	12.2 * (8.2–17.5)	14.4 ** (10.5–18.9)	6.5 (3.2–9.4)
	CD71	%	30.8 * (24.6–35.7)	34.5 * (22.7–39.4)	41.6 * (20.3–44.8)	24.5 (15.4–27.5)
	TLR2	%	9.1 ** (6.7–10.3)	8.8 ** (5.3–13.4)	8.5 ** (5.9–10.8)	3.0 (0.9–5.1)

The table summarizes the data of flow cytometry analysis surface marker expression on human peripheral blood cells (T- and B-lymphocytes) of healthy volunteers (n = 5). The time of incubation of fucoidans (100 µg/mL) with whole blood was 24 h. The data of the average percentage (%) of cells expressing CD molecules represent Me (median) and min-max; n = 5; * *p* < 0.05; ** *p* < 0.01 compared to the control, Wilcoxon-test.

**Table 4:** Compositions of fucoidans from the brown alga *Fucus evanescens.*

№	Characteristic of Fucoidans	Molecular Weight (kDa)	SO_3_Na^*^ (%)	Monosaccharide Composition (% mol)				
				Fuc	Gal	Xyl	Man	Glc
**F1**	Native fucoidan [35]	130–430	27.0	94.1	3.8	2.1	0	0
**F2**	Highly purified fucoidan [36]	130–400	28.1	94.4	3.5	2.1	0	0
**F3**	Product of enzymatic hydrolysis of fucoidan [6]	50.8	29.7	97.8	2.2	0	0	0

* % (Molecular weight) of the polysaccharide.

## Abbreviations

DC	dendritic cell
CD	cluster of differentiation
HBs AG	recombinant hepatitis B virus surface antigen
IFN-γ	interferon gamma
IG	immunoglobulin
IL	interleukin
MAbs	monoclonal antibodies
NK	natural killer cells
OVA	ovalbuminum
Th1, Th	T-helper1, 2
TNF-α	tumor necrosis factor alpha
